# Metabolite Markers for Characterizing Sasang Constitution Type through GC-MS and ^1^H NMR-Based Metabolomics Study

**DOI:** 10.1155/2019/8783496

**Published:** 2019-02-03

**Authors:** Eun-Ju Kim, Young-Shick Hong, Seung-Ho Seo, Seong-Eun Park, Chang-Su Na, Hong-Seok Son

**Affiliations:** ^1^School of Korean Medicine, Dongshin University, Naju, Jeonnam 58245, Republic of Korea; ^2^Division of Food and Nutrition, Chonnam National University, Gwangju 61186, Republic of Korea

## Abstract

Sasang constitutional medicine classifies human beings into four types based on their physical and psychological characteristics. Despite its potential value in achieving personalized medicine, the diagnosis of sasang constitution (SC) type is complex and subjective. In this study, gas chromatography–mass spectrometry and ^1^H nuclear magnetic resonance–based metabolic analyses were conducted to find maker metabolites in serum and urine according to different SC types. Although some samples were overlapped on orthogonal projection to latent structure discriminant analysis score plots, serum samples showed separation between different SC types. Levels of lactate, glutamate, triglyceride, and fatty acids in serum and glycolic acid in urine of Tae-Eum type were higher than those of So-Eum and So-Yang type. Fatty acids, triglyceride, and lactate levels were found to be metabolites related to body mass index, indicating that marker metabolites for the diagnosis of SC type could be associated with obese. However, Tae-Eum type showed higher lactate levels in serum than So-Yang type for both normal weight and overweight groups, suggesting that the contents of serum lactate might be dependent on the SC type regardless of body weight. These results suggest that metabolomics analysis could be used to determine SC type.

## 1. Introduction

Sasang constitutional medicine (SCM) is a Korean tailored medicine systematically theorized by Jema Lee (1837-1900). It classifies human beings into four constitutional types (Tae-Yang, Tae-Eum, So-Yang, and So-Eum) based on physical and psychological characteristics such as voice, face, and disease symptoms [1–5]. Traditional Korean medicine doctors claim that disease susceptibilities and drug response depend on sasang constitution (SC) type [6]. For example, it has been reported that the risk of diseases such as obesity and hyperlipidemia varies according to SC type [7, 8]. Furthermore, patients with the same disease could be differently treated according to SC types [9–11]. Despite its potential value in achieving personalized medicine, scientific evidence of SCM is still required to verify this. Nonetheless, SCM is recognized as a medical practice in Korea.

Since the diagnosis of SC type is determined by subjective view of Korean medicine doctor, the diagnostic process is subjective with unreliable aspects [12]. The classification of SC has been mainly done according to opinions of SCM specialists or using questionnaire sasang constitution classification (QSCC) [13, 14]. To determine SC type, expert knowledge of SCM is needed. Recently, sasang constitutional analysis tool (SCAT) has been developed to objectively measure factors such as face, voice, and body shape [15, 16]. Since SCAT also contains subjective factors, it is difficult to objectively determine SC types. To overcome this weakness, it is necessary to develop more objective indicators.

Metabolomics is systematic study of endogenous small metabolites [17–19]. Metabolomics approach has been used to diagnose diseases such as Alzheimer's disease, rheumatoid arthritis, and lupus for which the precise mechanism of disease development is unknown [20–22]. Serum and urine are useful body fluid samples that can be conveniently collected for diagnosis purpose since they contain various metabolites related to diseases [23]. Recently, Kim et al. [12] have reported some biomarkers for characteristics of SC using metabolomics combined with lipidomics in a pilot study. Except for their study, no other study has used metabolites of body fluids for the classification of SC type. Multiple complementary analytical platforms are needed for the detection of more metabolites. Thus, the objective of the present study was to find metabolite marker in serum and urine according to different SC types through metabolomics approach using gas chromatography–mass spectrometry (GC-MS) and ^1^H nuclear magnetic resonance (^1^H NMR).

## 2. Materials and Methods

### 2.1. Classification of SC and BMI

A total of 107 subjects between 20 and 29 years of age were recruited. Subjects who voluntarily applied to be participants of this study were assessed for the classification of SC type using SCAT developed by the Korea Institute of Oriental Medicine (KIOM). Individual SC type was first determined based on information of body shape, voice, and face and questionnaire information obtained from SCAT. Each SC type was confirmed by SCM specialist. When diagnostic results between SCAT and SCM specialist were different, subjects were excluded from further analysis. A total of 14 participants were excluded from the study because SC type diagnosis between SCAT and SCM specialist was inconsistent.

### 2.2. Sample Collection and Preparation

Blood and urine samples were collected from volunteers in a local Health Center (Naju, Jeonnam, Korea). These subjects had fasted 12 h prior to collection of blood samples. Blood samples were transferred to serum separator tubes and left at room temperature for complete coagulation. Serum sample preparation protocol for GC-MS analysis was similar to that described in previous studies [24, 25] with slight modifications. Briefly, 100 *μ*L of serum samples was thawed at 4°C and mixed with 300 *μ*L of methanol to precipitate protein. The mixture was then vortexed for 15 s. After centrifuging the sample at 13,000 rpm for 5 min at 4°C, 300 *μ*L of supernatant was freeze-dried. For ^1^H NMR analysis, 300 *μ*L of serum samples was thawed at 4°C and centrifuged at 13,000 rpm for 15 min at 10°C. Next, 200 *μ*L of supernatant was taken for NMR analysis.

Urine samples were collected on ice and stored at −80°C deep freezer until experiment. Urine sample preparation protocol for GC-MS analysis was conducted according to the method described by Kałużna-Czaplińska et al. [26]. Briefly, urease (40 units) was added to 200 *μ*L of urine followed by incubation at 37°C for 20 min to remove urea. Next, 800 *μ*L of methanol was added to precipitate protein. The mixture was then vortexed, centrifuged, and freeze-dried as described above.

### 2.3. ^1^H NMR Analysis

A volume of 200 *μ*L serum was mixed with 400 *μ*L of saline solution containing 10% D_2_O. Samples were centrifuged at 13,000 rpm for 15 min at 10°C and then 550 *μ*L supernatants were transferred into 5 mm NMR tubes for NMR analysis. ^1^H NMR spectra were acquired at 298 K using a Bruker Avance 700 NMR spectrometer (Bruker Biospin, Rheinstetten, Germany) and using the water-presaturated standard one-dimensional Carr-Purcell-Meiboom-Gill (CPMG) pulse sequence. Free induction decays (FID) of serum was collected with 128 transients into 32 K data points using a spectral width of 10 kHz with relaxation decay of 2 s and relaxation time of 100 ms.

### 2.4. GC-MS Analysis

Freeze-dried serum and urine samples were dissolved in 100 *μ*L of O-methoxamine hydrochloride in pyridine (15 mg/mL). After vortexing each sample for 3 min at room temperature, 100 *μ*L of* N,O*-bis-(trimethylsilyl)-trifluoroacetamide (BSTFA) containing 1% trimethylchlorosilane (TMCS) was added to each sample and incubated at 70°C for 1 h. Next, 600 *μ*L of methyl stearate (10 ppm in heptane) was added as an internal standard. After centrifuging samples at 14,000 rpm for 5 min, 600 *μ*L of supernatant was transferred to a 1.5 mL glass vial for GC-MS analysis. GC-MS analysis conditions were the same as those described in our previous study [27].

### 2.5. Data Processing

All NMR spectra were manually corrected for phase and baseline distortions and then converted to ASCII format. ASCII format files were then imported into MATLAB R2010b (MathWorks, Inc., Natick, MA, USA). All spectra were aligned by the icoshift method [28] and normalized using probabilistic quotient normalization method [29]. The water regions from d4.7 to 4.9 ppm were excluded prior to the spectral normalization.

GC-MS metabolite profiles were converted to CDF format and placed at XCMS website (https://xcmsonline.scripps.edu) for feature detection, retention time correction, baseline correction, and peak alignment. A default Centwave method for GC Single Quadruple was selected with the following parameters: signal/noise threshold, 2; mzdiff, 0.1; integration methods, 1; prefilter peaks, 3; prefilter intensity, 10000; mzwid, 0.25; minfrac, 0.5; and bandwidth, 3. Next, values were corrected by subtracting the average of the blank sample at each feature. Feature intensities were normalized according to the intensity of methyl stearate (internal standard) prior to multivariate statistical analyses. Identification of metabolites was conducted based on fragmentation patterns of NIST library, RI value, and other researcher's experimental data using the same method.

### 2.6. Multivariate Statistical Analysis

Resulting data were imported into SIMCA-P 15.0 version software package (Umetrics, Umea, Sweden) for multivariate statistical analysis such as Principal Component Analysis (PCA). Orthogonal Projection to Latent Structure Discriminant Analysis (OPLS-DA) was also performed to extract information on discriminant compounds [30]. To validate the supervised model, permutation tests were conducted with 200 iterations. A visual assessments of OPLS-DA loading or coefficient derived from ^1^H NMR spectra were performed with color-coded correlation coefficient of variables using an in house developed script for MATLAB.* R*^2^*X* and* R*^2^*Y* were used to explain variability of variables while *Q*^2^ was used to indicate model predictive capability. Sample distinction variables with high variable importance in projection (VIP) value (VIP > 1.0) and low* p*-value (*p* < 0.05) were selected for statistical analysis. All statistical analyses were performed using SPSS version 22.0 software package (SPSS Inc., Chicago, IL, USA). T-test was performed to compare the relative amount (peak intensity) of identified metabolites between the two groups. Analyses of variance (one-way ANOVA) followed by Duncan multiple-range test was also performed for examination of group differences.

### 2.7. Ethical Approval

This study was approved by the Institutional Review Board (IRB) of the Ministry of Health and Welfare (approval number: P-01-201710-30-002).

## 3. Results

### 3.1. SC Classification

General characteristics and weight distribution of subjects by SC type are summarized in Table 1. Tae-Yang, Tae-Eum, So-Yang, and So-Eum were classified as 1, 43, 20, and 29, respectively. Tae-Yang is extremely uncommon constitution for population [31]. In this study, only one subject was diagnosed as Tae-Yang type and was therefore excluded from further analysis.

Body mass index (BMI) was calculated by the formula of weight (kg)/height (m^2^). Subjects were classified into underweight (BMI < 18.5), normal weight (BMI, 18.5-22.9), overweight (BMI, 23.0-24.9), and obese (BMI > 25.0), which were determined by the WHO BMI criteria for adult Asians [32, 33].

### 3.2. Metabolic Differentiation of Serum by ^1^H NMR

**Figure 1:**
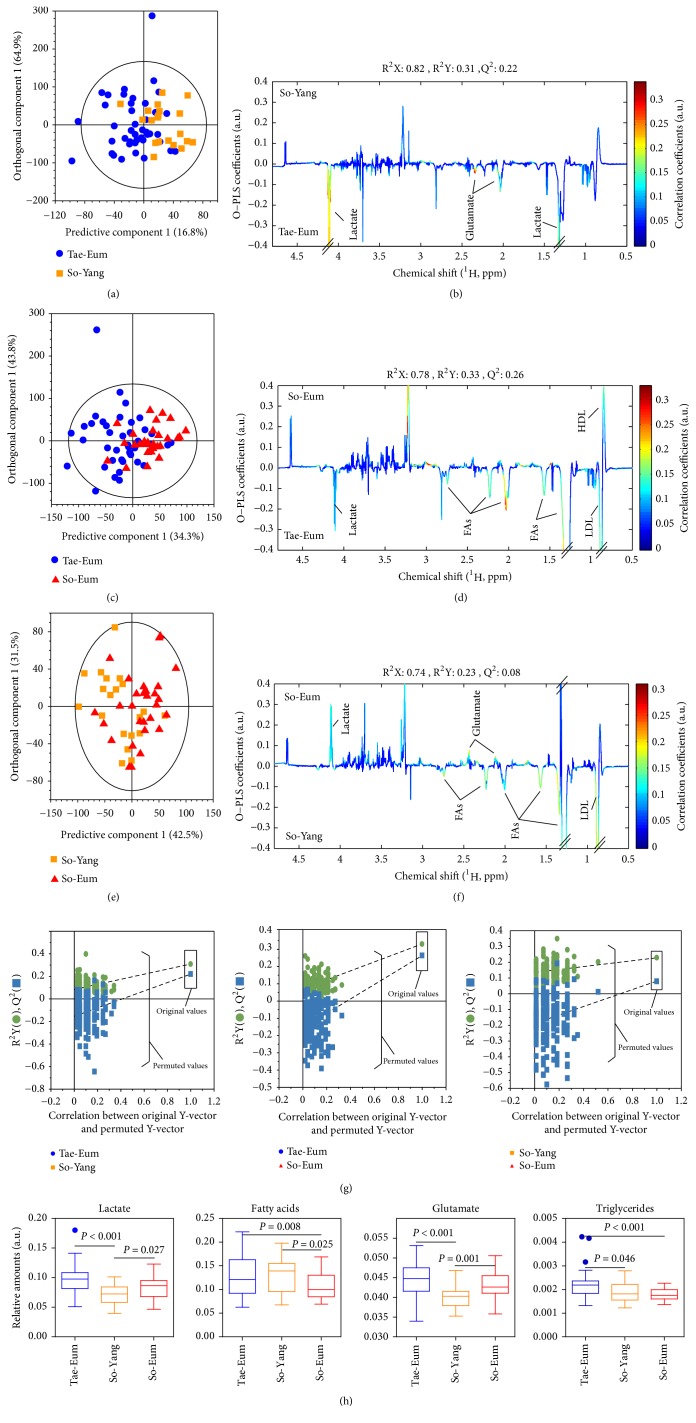
OPLS-DA score (a, c, and e) and OPLS-DA loading (b, d, and f) plots derived from ^1^H NMR spectra of serum samples for pairwise comparison of metabolic difference between Tae-Eum and So-Yang (a and b), Tae-Eum and So-Eum (c and d), and So-Yang and So-Eum (e and f), demonstrating metabolic differences in serum between SC types. Reliabilities and predictabilities of OPLS-DA models were indicated by* R*^2^*X* and *Q*^2^ (g), respectively. HDL, high-density lipoprotein; LDL, low-density lipoprotein; FAs, fatty acids. Panels (h) show scatter dot plots of identified metabolites.

Whole ^1^H NMR spectra of serum samples were applied to multivariate statistical analysis such as PCA and OPLS-DA. However, samples were not separated in the PCA score plot among SC types. To maximize separation, OPLS-DA models for supervised pattern recognition were further applied between two SC type samples (See Figure 1). OPLS-DA score plots of samples showed a separation pattern by PC1 between different SC types, indicating that metabolite profiles of serum could be different by SC type (See Figures 1(a), 1(c), and 1(e)). A permutation test (200 permutations) was performed to verify the OPLS-DA model. Through a permutation test for the OPLS-DA model, values of *Q*^2^ and *R*^2^ were confirmed to be higher than their original values, proving the validity and suitability of this model (see Figure 1(g)). Pairwise OPLS-DA models between SC types were generated to identify metabolites in serum according to SC type. In the comparison between Tae-Eum and So-Yang types, levels of lactate and glutamate were higher in serum samples of Tae-Eum type than those in So-Yang type (see Figure 1(b)). Levels of high-density lipoprotein (HDL) in serum samples of So-Eum type were higher than those in Tae-Eum type whereas levels of lactate, fatty acids (FAs), and low-density lipoprotein (LDL) were lower in serum samples of So-Eum type (see Figure 1(d)). Levels of lactate and glutamate in serum samples of So-Eum type were higher than those in So-Yang type whereas levels of FAs and LDL were lower in serum samples of So-Eum type than those in So-Yang type (see Figure 1(f)). Quantitative comparisons of serum lactate, FAs, glutamate, and triglycerides among SC types are shown in Figure 1(h). These results are similar to findings of Kim et al. [12], showing significantly higher levels of FAs but lower levels of glutamate in serum samples of So-Yang type than of other types. These metabolites might be potential biomarkers for the diagnosis of SC type.

### 3.3. Metabolic Differentiation of Serum by GC-MS

**Figure 2:**
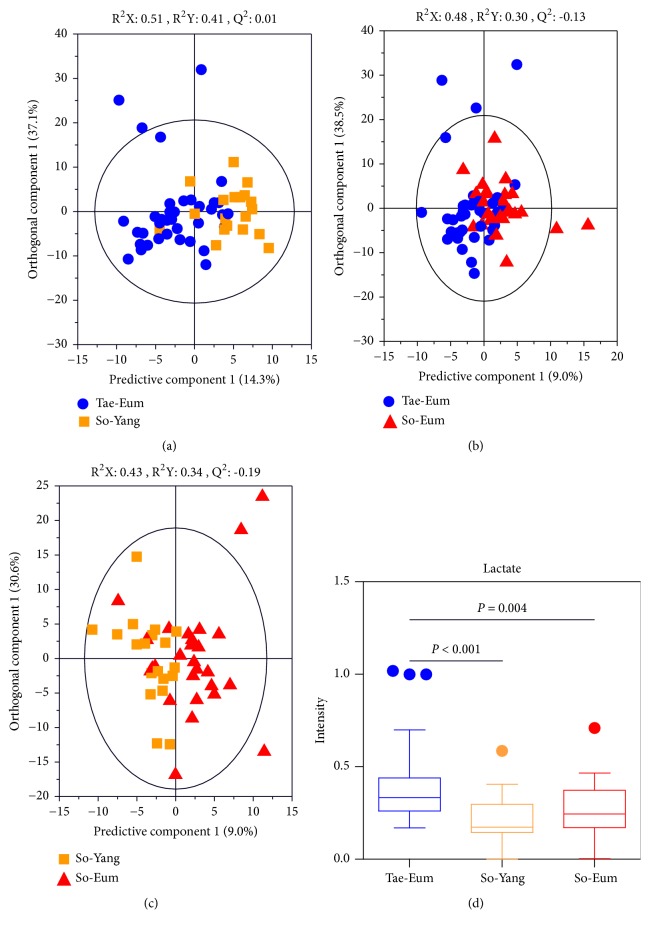
OPLS-DA score plots derived from GC-MS data of serum samples between Tae-Eum and So-Yang (a), Tae-Eum and So-Eum (b), and So-Yang and So-Eum (c), demonstrating metabolic differences in serum samples between SC types. Panel (d) shows relative amounts of serum lactate contributing to differentiation in the OPLS-DA model (VIP > 1.0 and* p* < 0.05).

To investigate metabolic differences in serum according to SC types, PCA and OPLS-DA models were generated using GC-MS data. Similar to the results of ^1^H NMR analysis, samples were not separated in the PCA score plot between SC types. OPLS-DA score plots of serum samples between two SC types are shown in Figure 2. Although some samples overlapped on the OPLS-DA score plot, some samples showed a separation pattern between groups (see Figures 2(a)–2(c)). To investigate which metabolites were responsible for the separation of the two groups, VIP score was determined. Based on a VIP score > 1.0 from OPLS-DA with* p* < 0.05 in two-tailed Student's* t*-test, the levels of lactate in serum samples from Tae-Eum type subjects were significantly higher than those from So-Yang and So-Eum type subjects (see Figure 2(d)). These results are consistent with ^1^H NMR analysis results in the current study. In comparison between So-Eum and So-Yang types, no metabolites showed significant differences.

### 3.4. Metabolic Differentiation of Urine by GC-MS

**Figure 3:**
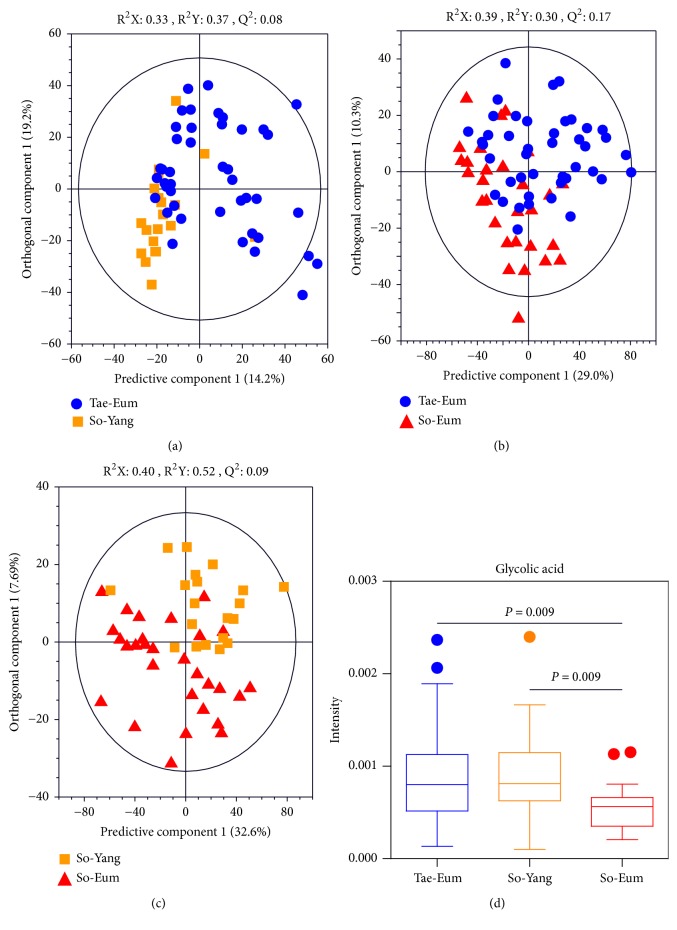
OPLS-DA score plots derived from GC-MS data of urine samples between Tae-Eum and So-Yang (a), Tae-Eum and So-Eum (b), and So-Yang and So-Eum (c), demonstrating poor metabolic differences in urine samples between SC types. Panel (d) shows relative amounts of urinary glycolic acid contributing to differentiation in the OPLS-DA model (VIP > 1.0 and* p* < 0.05).

To investigate metabolic differences in urine according to SC types, OPLS-DA models were generated using GC-MS data. In the OPLS-DA score plot, urine samples were not fully separated between SC types (see Figures 3(a)–3(c)). The levels of glycolic acid in urine of Tae-Eum type were higher than those of So-Eum and So-Yang type (see Figure 3(d)).

### 3.5. Comparison of Metabolites by BMI

**Figure 4:**
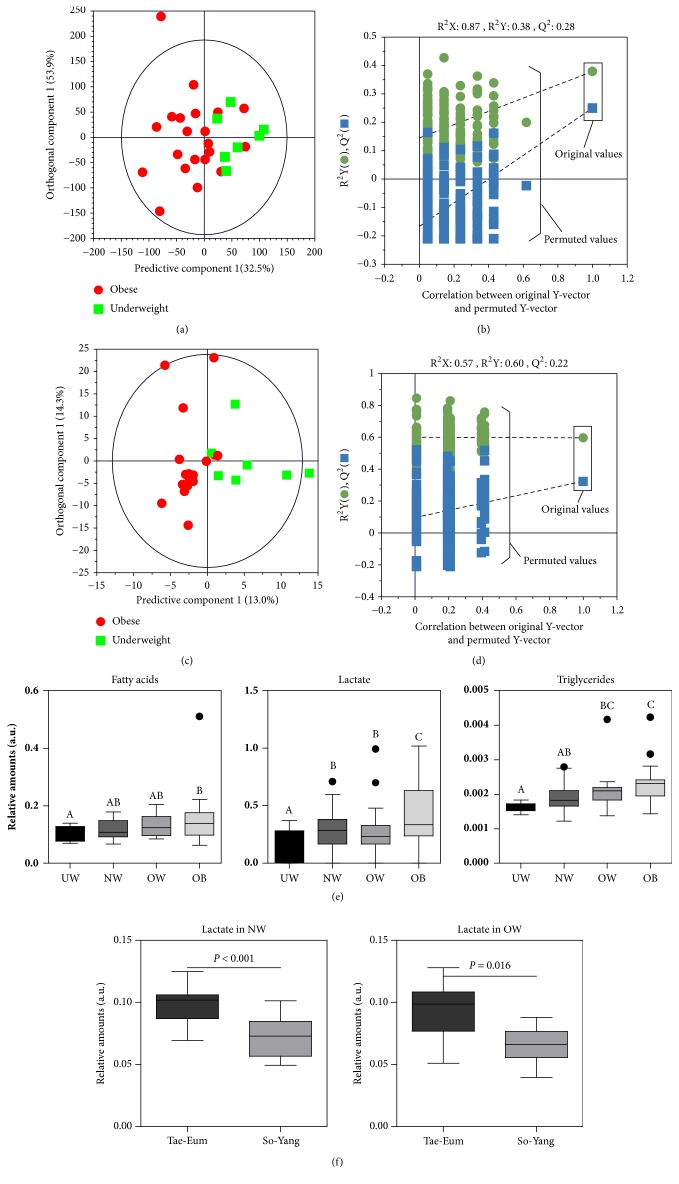
OPLS-DA score plots derived from ^1^H NMR spectra (a) and GC-MS data (c) of serum samples between underweight and obese people, and validations of the models by a permutation test repeated 200 times (b and d). Panel (e) shows a comparison of metabolites that are significantly different according to BMI regardless of SC type. Subjects were classified into underweight (BMI < 18.5, n=8), normal weight (BMI, 18.5-22.9, n=44), overweight (BMI, 23.0-24.9, n=20), and obese (BMI > 25.0, n=20). Panel (f) shows difference in serum lactate contents between Tae-Eum and So-Yang types with normal weight and overweight, demonstrating metabolic dependence of serum lactate level on SC types. Values are presented as mean ± SD. Different alphabets indicate significant differences among groups by Duncan's test at* p* < 0.05. UW, underweight; NW, normal weight; OW, overweight; OB, obese.

In this study, height was not significantly different according to SC type. However, weight and BMI of Tae-Eum type subjects were significantly different from those of other SC types (see Table 1). Tae-Eum type subjects are well known to be more obese than subjects with other types [34]. Chae et al. [14] have also reported that Tae-Eum type subjects have relatively higher BMI. Interestingly, in the present study, all obese people were of Tae-Eum type while almost all underweight people were of So-Eum type, suggesting that BMI might be related to SC type classification. To find different metabolites dependent on BMI, OPLS-DA was performed with serum samples of people determined to be obese or underweight. OPLS-DA score plots derived from ^1^H NMR and GC-MS data of serum samples from obese and underweight people are shown in Figure 4. There was clear separation between samples of obese and underweight people in both score plots (see Figures 4(a) and 4(c)). OPLS-DA models were validated by permutation tests repeated 200 times (see Figures 4(b) and 4(d)). The levels of FAs, triglyceride (TG), and lactate, which were found to be significantly higher in Tae-Eum type, were confirmed to be metabolites related to BMI, indicating that metabolite markers for the diagnosis of SC could be associated with obese (see Figure 4(e)). To exclude effects of BMI, samples of subjects with BMI were taken and compared for differences in metabolites according to SC type. The statistics of similar BMI between Tae-Eum and So-Yang are shown in Table S1. In comparison with samples with similar BMI value (normal weight and overweight), Tae-Eum type showed higher lactate levels in serum than So-Yang type for both normal weight and overweight groups (see Figure 4(f)). Although BMI of Tae-Eum type in normal weight was higher than that of So-Yang type (*p* = 0.015), these results suggest that the contents of serum lactate might be dependent on the SC type regardless of body weight.

## 4. Discussion

According to SCM, disease susceptibilities and drug response vary with SC types. Thus, accurate and objective diagnosis of SC type is essential in clinical SCM practice. Without an objective diagnosis of SC, the SCM theory could be difficult to be developed. However, the current diagnostic system has no standardized method based on clear objective evidence. So far, attempts to achieve standardization have mainly focused on four measurement factors: face, voice, body shape, and questionnaires [16]. Data collection for SC type classification in SCAT also includes the following four factors: facial photographs, body measurements, voice recordings, and SCM questionnaires [35]. Nevertheless, it is difficult to conclude that any individual factor contributes significantly to the classification of SC.

Generally, Tae-Eum type subjects have physical traits of higher BMI and bigger chest circumference compared to So-Yang and So-Eum types [14, 36, 37]. Several studies have suggested that SC could be associated with some chronic diseases. According to Jang et al. [38], Tae-Eum type might be significantly and independently associated with abdominal obesity. Although there is no clear study on the relationship between energy metabolism and SC type, large body shape and high BMI are representative features of Tae-Eum type [9]. Likewise, in the current study, Tae-Eum type subject had significantly higher body weight and BMI than So-Yang and So-Eum types. In addition, high levels of lactate, TG, and FAs in serum samples of obese people were also observed, suggesting that metabolites could be used as biomarkers to explain the proclivity of Tae-Eum type to progress to obesity. Some previous studies have reported that genetic polymorphisms of several obesity-related genes are associated with SC types. Cha et al. [39] have reported that obesity is associated with genes FTO and MC4R only in Tae-Eum type subjects. Polymorphisms of IL-1*α* and IL-1*β* are also associated with obese women of Tae-Eum type [40, 41]. However, the precise mechanism involved in the influence of these gene polymorphisms is currently unknown. A systematic review on studies related to genetic characteristics of SC types has also indicated that there is no conclusive genotype related to SC type [42].

In the current study, serum levels of TG, FAs, and lactate were found to be significantly higher in Tae-Eum type subjects. TG and FAs are well known to be related to BMI. Many studies have shown that lactate is also an important obesity-associated metabolite. Obese subjects can have higher lactate release due to their larger adipose mass [43]. Lactate is also an important precursor of gluconeogenesis. It can be point of perturbation in obese subjects [44]. However, when people with similar BMI value were compared, the levels of serum lactate were significantly higher in Tae-Eum type subjects than in So-Yang type subjects (see Figure 4(f)). These results suggest that there may be differences in blood metabolites depending on SC types. Cho et al. [45] have reported that the incidence of type 2 diabetes mellitus is much higher in Tae-Eum type subjects. As plasma lactate level is strongly associated with diabetes [46], high prevalence of diabetes in Tae-Eum type subjects might be associated with high levels of lactate.

The fact that SC types are determined by physiologic and physical characteristics indicates that there might be differences at molecular level according to SC types. This study is a new attempt to find objective biomarkers for SC diagnosis using metabolomics approach. Tae-Eum type showed higher lactate levels in serum than So-Yang type for similar BMI groups, suggesting that the levels of serum lactate might be dependent on the SC type regardless of BMI. These results suggest that lactate levels could be used as a potential biomarker for SC type classification. However, the number of samples used in this study was insufficient to identify each SC type-associated biomarkers. Further metabolomics study involving a lot of people is required to find objective indicators for SC diagnosis. The relationship between molecular functions and physiologic/physical characteristics of SC type also should be elucidated in the future.

## Figures and Tables

**Table 1:** Anthropometric and clinical characteristics of subjects by SC type.

Variable	All	Tae-Eum	So-Yang	So-Eum
General characteristics				
Number (N)	93	43	20	29
Female (%)	27.0	16.3	40.0	41.4
Age (years)	26.2 ± 6.3	26.7 ± 7.6	26.2 ± 4.6	25.5 ± 5.0
Height (cm^2^)	171.5 ± 8.0	173.2 ± 7.3	169.3 ± 8.8	170.6 ± 8.4
Weight (kg)	66.5 ± 13.6	74.0 ± 13.3^a(1)^	60.8 ± 9.9^b^	59.1 ± 10.2^b^
BMI (kg/m^2^)	22.4 ± 3.4	24.6 ± 3.5^a^	21.1 ± 2.0^b^	20.2 ± 1.9^b^
Weight distribution (%)				
Underweight	8.6	-	5.0	24.1
Normal weight	47.3	27.9	65.0	65.5
Overweight	22.6	25.6	30.0	10.3
Obese	21.5	46.5	-	-

^(1)^ Different letters indicate significant differences between groups (*p* < 0.05). Values are presented as mean ± standard deviation.

BMI: body mass index.

## Data Availability

The research article data used to support the findings of this study are available from the corresponding authors upon request.

## Abbreviations

BMI: Body mass index
FAs: Fatty acids
GC-MS: Gas chromatography–mass spectrometry
HDL: High-density lipoprotein
LC-MS: Liquid chromatography–mass spectrometry
LDL: Low-density lipoprotein
NMR: Nuclear magnetic resonance
OPLS-DA: Orthogonal projection to latent structure discriminant analysis
PCA: Principal component analysis
QSCC: Questionnaire Sasang constitution classification
SC: Sasang constitution
SCAT: Sasang constitutional analysis tool
SCM: Sasang constitutional medicine
TG: Triglyceride
VIP: Variable important in projection.

## Supplementary Materials

Supplementary Materials Table S1: BMI comparison between Tae-Eum and So-Yang in normal weight and overweight.
