# Supplementary material for: Metabolite Markers for Characterizing Sasang Constitution Type through GC-MS and 1H NMR-Based Metabolomics Study
Source: Evid Based Complement Alternat Med. 2019 Feb 3;2019:8783496. doi: 10.1155/2019/8783496 (PMC6378031; doi:10.1155/2019/8783496)
Supplement: Supplementary Materials — Table S1: BMI comparison between Tae-Eum and So-Yang in normal weight and overweight. [file 8783496.f1.docx]

Supplementary Material

**Metabolite Markers for Characterizing Sasang Constitution Type through GC-MS and ^1^H NMR-based Metabolomics Study**

**Table of contents:**

Table S1: BMI comparison between Tae-Eum and So-Yang in normal weight and overweight.

| BMI | Tae-Eum | So-Yang | *p*-value |
| --- | --- | --- | --- |
| Normal weight(N) | 21.26±0.89(13) | 20.24±1.13(14) | 0.015 |
| Overweight(N) | 23.15±0.46(12) | 23.47±0.60(5) | 0.231 |

Values are present as mean ± standard deviation.

N: Number of people.
